# Umbrella Refinement of Ensembles—An Alternative View of Ensemble Optimization

**DOI:** 10.3390/molecules30112449

**Published:** 2025-06-03

**Authors:** Johannes Stöckelmaier, Tümay Capraz, Chris Oostenbrink

**Affiliations:** 1 Institute of Molecular Modeling and Simulation (MMS), BOKU University, 1190 Vienna, Austria; johannes.stoeckelmaier@boku.ac.at; 2 European Molecular Biology Laboratory (EMBL), 69117 Heidelberg, Germany; 3 Christian Doppler Laboratory Molecular Informatics in the Biosciences, BOKU University, 1190 Vienna, Austria

**Keywords:** ensemble reweighting, maximum entropy, conformational ensemble

## Abstract

The elucidation of protein dynamics, especially in the context of intrinsically disordered proteins, is challenging and requires cooperation between experimental studies and computational analysis. Molecular dynamics simulations are an essential investigation tool but often struggle to accurately quantify the conformational preferences of flexible proteins. To create a quantitatively validated conformational ensemble, such simulations may be refined with experimental data using Bayesian and maximum entropy methods. In this study, we present a method to optimize a conformational ensemble using Bayes’ theorem in connection with a methodology derived from Umbrella Sampling. The resulting method, called the Umbrella Refinement of Ensembles (URE), reduces the number of parameters to be optimized in comparison to the classical Bayesian Ensemble Refinement and remains methodologically suitable for use with the forward formulated Kullback–Leibler divergence. The method is validated using two established systems, an alanine–alanine zwitterion and the chignolin peptide, using nuclear magnetic resonance data from the literature.

## 1. Introduction

Obtaining a complete understanding of protein folding remains one of the key challenges in structural biology. Despite the vast improvements obtained in the last decade, the transition from a loose biopolymer into a functional enzyme is not yet fully understood. Since the mid-20th century, the lock-and-key theory describing the structure–function relationship of proteins has been a foundation of pharmaceutical research and the understanding of biochemical processes [1,2]. This theory tightly connects geometry with biological function, which is only possible due to the reproducible folding of polypeptides into structured proteins. In the new millennium, additional scientific discoveries started to weaken this theory, replacing it with more nuanced theories. It was shown that a substantial share of proteins feature intrinsically disordered regions [3] and the elucidation of protein variability and dynamics has become a major topic of scientific interest.

Proteins can be classified into different levels of overall structural stability [4]. Structured proteins show a well-defined three-dimensional geometry and remain thermodynamically stable under ambient conditions while structurally more diverse proteins contain intrinsically disordered regions (IDRs). If the entire protein takes on many diverse conformations, it is classified as an intrinsically disordered protein (IDP) [5]. IDPs and proteins containing IDRs are often physiologically active and seem to be key in the understanding of diseases like Alzheimer’s [6], Parkinson’s [7,8], and cancer [9].

### 1.1. The Conformational Ensemble

An advanced understanding of a disease mechanism is a critical precondition of developing new generations of pharmaceutics [10,11]. Due to the involvement of flexible proteins in many such mechanisms, a proper representation of protein structure and dynamics is expected to improve the prospects of a medical breakthrough [12,13,14]. In silico studies typically involve molecular dynamics (MD) simulations to predict the properties and dynamics of molecules. MD leverages the physical laws of motion to calculate the movements and interactions of biomolecules in and with their surroundings. The calculated protein behavior is governed by an accurate energy function, which is an approximation of the interactions between atoms and molecules that is more correctly described by quantum mechanics. The interactions described by the energy function are parametrized by the force-field, defining the system’s characteristics [15,16,17]. The regular sampling of conformations during simulation creates a trajectory, a representation of the molecular motion over time. A sufficiently long trajectory represents the most basic form of a conformational ensemble [18,19,20], describing the protein’s structure and dynamics. A conformational ensemble is a set of individual, geometrical structures, each describing one possible conformer with an associated statistical weight [21]. The statistical weight describes the importance of an individual structure in the ensemble, which is uniformly 1/N in the case of a regularly sampled, unbiased MD simulation. Conformational clustering [22,23] is often used to compress the size of the initial ensemble, binning similar conformers into one cluster with one representative conformer and setting the statistical weight of every cluster proportional to the cluster size.

Molecular dynamics simulation has seen substantial methodological improvements over the past decades, making it an established and widely used technology [17]. Despite this success, the simulation of flexible proteins and molecular systems that involve multiple accessible conformations remains challenging [24,25,26]. For these systems, represented by multiple minima in the potential energy landscape, the appropriate sampling of these minima involves crossing energy barriers. If these transitions are infrequent because of the high barriers, appropriate sampling the relative occurrence of the minimum energy conformers remains challenging and will require extremely long simulation times. The currently accessible time scales of MD simulations allow for direct observations of many of these switches, although not always frequently enough to obtain statistically robust weights of the (clustered) conformational ensemble.

Similarly, systems with frequent transitions between many conformers, as is the case with disordered proteins, remain prone to error. Most force-fields are designed to accurately reproduce the geometrical ground states and potential energy minima of a biophysical system but focus less on describing the energy barriers which govern the transition from one conformer to the next. The complex, multifunneled potential energy landscapes of IDPs with multiple, often shallow, minima [27,28] and flatter parts that can span multiple conformations [29] make simulations of IDPs difficult. These kinds of energy surfaces allow for switches between different conformations at ambient temperatures where small inaccuracies in the description of the energy barrier by the force-field may lead to non-ideal statistical weights for the resulting ensemble.

### 1.2. Reweighting of Ensembles

These challenges, connected to the flexibility of proteins, lead to inaccuracies that may change the predicted properties and introduce unexpected errors. To validate the simulation and possibly correct inaccuracies, the computational findings should be compared to experimental studies. One group of methods used to systematically refine simulated properties with experimental data is reweighting methods [30,31,32,33]. These methods compare the conformational ensemble obtained by computational means with experimental data to adjust the statistical weight of each conformer such that the refined ensemble is in sufficiently good agreement.

To compare conformational ensembles with experimental data, the same observables as those measured in the experiment must be calculated. Observables are typically calculated for each conformer in the ensemble individually, as many observables are sensitive to conformational changes [34]. In contrast, experimental measurements almost always represent averages of the entire molecular population. Therefore, measured observables do not represent single conformations, but an average of the entire conformational ensemble [35,36,37,38]. It is thus necessary to compute an ensemble average from the individually calculated single conformers to provide a valid comparison between them and the experimental data.

With most types of observables, a weighted average over the simulation trajectory is calculated. Equation (1) shows such an averaging where $O_{t}^{calc}$ is the calculated observable of conformer *t*. Symbol $w_{t}$ represents the statistical weight of conformer *t* and *N* is the total number of conformations in the ensemble. The angular brackets indicate an ensemble average.

(1) $$\langle O^{calc}\rangle =\sum_{t=0}^{N}\left(w_{t}*O_{t}^{calc}\right)$$

Special care needs to be taken with residual dipolar couplings (RDCs) and nuclear Overhauser effects (NOEs), where different averaging schemes are required. The intensity of the NOE signal is dependent on the distance between the relevant proton pair and weakens proportional to the third or sixth power of the distance, depending on the tumbling time, the internal motion, and the size of the molecule [39]. Pairs closer than 3 Å provide strong signals while the limit of detection is reached with pairs 6 Å apart. This means that a small number of conformers with a short distance between the proton pair can have a dominating influence on the NOE signal. To calculate the ensemble average for interatomic distances that are to be compared to NOE-derived bounds, either $r^{-3}$ or $r^{-6}$ averaging (Equation (2)) is used.

(2) $$\langle O_{NOE}^{calc}\rangle ={\left[\sum_{t=0}^{N}w_{t}*{\left(O_{NOE}^{calc}\right)}_{t}^{-6}\right]}^{-1/6}$$

The statistical weight $w_{t}$ is interpreted as the probability that conformer *t* occurs, which leads to the condition that the sum of all weights needs to be one:

(3) $$\sum_{t=0}^{N}w_{t}=1.0$$

**Figure 1:**
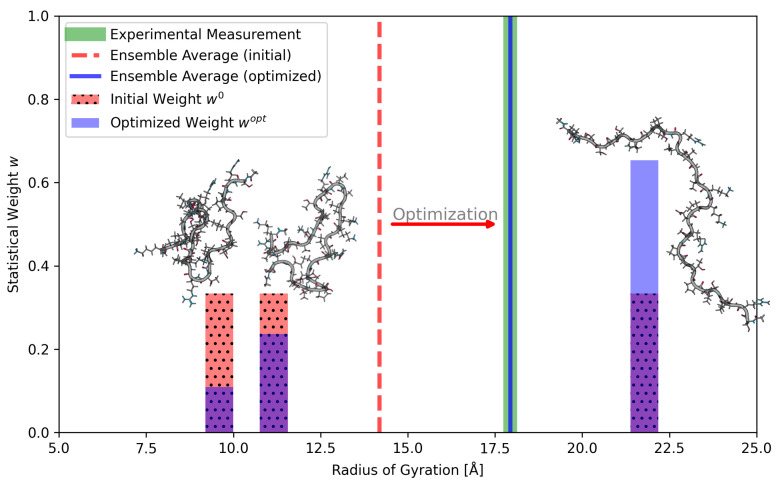
This hypothetical example demonstrates the reweighting of a very small ensemble of three conformers. Due to the lack of initial knowledge, it is unknown if any of the three conformers are dominant within the ensemble; thus, we assume uniform initial weights (red bars, dotted). Using these initial weights, the ensemble average of the radius of gyration is calculated, with a value of around 14 Å (dashed red line). From a hypothetical experiment, we know that the true expected radius of gyration is higher, at around 18 Å (green vertical line). To bring the calculated ensemble average in agreement with the experiment, the statistical weights of the ensemble must be adjusted. This process gives more weight to the extended structure and reduces the impact of the two compact ones. The adjusted weights (blue bars) then also provide a new optimized ensemble average (blue vertical line), which is now in much better agreement with the experiment. If applied to larger systems, the adjustment of statistical weights becomes non-trivial and is typically solved automatically with a reweighting algorithm, taking multiple observables into account.

Figure 1 shows a hypothetical example to visualize ensemble averaging and the concept of reweighting. In the example, one property (the radius of gyration) of the molecule is known and an ensemble of three needs to be adjusted to reproduce the expected radius of gyration. During its application in a real system, the general process of reweighting works similarly, but with a higher number of observables and a much larger ensemble.

In this study, we propose an ensemble reweighting strategy that leverages the solid theoretical foundation of Umbrella Sampling [40] to optimize the conformational ensemble. We first describe the method and discuss the regularization of optimization strength, and then validate the method using two systems.

## 2. Umbrella Refinement of Ensembles

Enhanced sampling methods are used in molecular dynamics to flatten or bias the potential energy surface (PES) of the system and to allow for the more complete or more focused sampling of the conformational space. By adding a biasing potential $V^{bias}$ while running the simulation, the PES is changed. Accordingly, these changes are embedded in the trajectory obtained from sampling the PES, making later adjustments complicated. The ideal PES would correspond to the real natural energy function $V^{nat}$, which is unknown but assumed to exist. One can attempt to define a biasing potential that transforms the PES of the force-field ($V^{FF}$) into the natural but unknown $V^{nat}$.

(4) $$V^{nat}\left(r\right)=V^{FF}\left(r\right)+V^{bias}\left(r\right)$$

The trajectories obtained using biased simulations to approximate $V^{nat}$ are specific to the chosen $V^{bias}$, an often undesirable effect as they no longer represent the Boltzmann ensemble of the force-field. Moreover, the definition of the appropriate $V^{bias}$ before running the simulation is non-trivial, potentially promoting misleading results.

Alternatively, a posteriori reweighting schemes can be applied to adjust an existing ensemble towards the natural PES after the simulation is finished to improve its agreement with the experimental data. In the context of a biasing potential, we can estimate the ensemble properties of the system for the natural PES from the ensemble averages of the force-field, as introduced by Torrie and Valleau [40]:

(5) $$\begin{array}{cc} {\langle O^{calc}\rangle }_{nat} & =\frac{\int O^{calc}*\exp\left(-V^{nat}/k_{B}T\right)dr}{\int \exp(-V^{nat}/k_{B}T)dr}\\ \; & =\frac{{\langle O^{calc}*\exp\left(\frac{-V^{bias}}{k_{B}T}\right)\rangle }_{FF}}{{\langle \exp\left(\frac{-V^{bias}}{k_{B}T}\right)\rangle }_{FF}} \end{array}$$

where $k_{B}$ is the Boltzmann’s constant, *T* is the absolute temperature, and the brackets 〈〉 indicate the ensemble average, with the subscript indicating the PES for which it is appropriate. From an MD simulation, an initial ensemble with associated weights $w^{0}$ is obtained. To reweight a property $O^{calc}$ from such an ensemble, Equation (5) can be rewritten to be used with N discrete conformations (or: samples) (Equation (6)).

(6) $${\langle O^{calc}\rangle }_{nat}=\frac{\sum_{t=0}^{N}w_{t}^{0}*O^{calc}*\exp\left(\frac{-V^{bias}}{k_{B}T}\right)}{\sum_{t=0}^{N}w_{t}^{0}*\exp\left(\frac{-V^{bias}}{k_{B}T}\right)}$$

As an alternative to Umbrella Sampling, the property ${\langle O^{calc}\rangle }_{nat}$ can also be calculated using a weighted ensemble average if the quantitatively correct weights $w^{opt}$ are known. This is typically not the case but allows us to combine Equation (6) with Equation (1). This leads to Equation (7), which shows the two approaches used to calculate the expectation value of the observable $O^{calc}$ under the assumption of a natural PES.

(7) $$\sum_{t=0}^{N}\left(O_{t}^{calc}*w_{t}^{opt}\right)={\langle O^{calc}\rangle }_{nat}=\frac{\sum_{t=0}^{N}w_{t}^{0}*O_{t}^{calc}*\exp\left(\frac{-V^{bias}}{k_{B}T}\right)}{\sum_{t=0}^{N}w_{t}^{0}*\exp\left(\frac{-V^{bias}}{k_{B}T}\right)}$$

From Equation (7), the relation between optimal weights $w^{\mathrm{opt}}$ and force-field-derived weights $w^{0}$ becomes evident:

(8) $$\begin{array}{c} w_{t}^{opt}=\frac{w_{t}^{0}}{Z}\exp\left(\frac{-V_{t}^{bias,opt}}{k_{B}T}\right)\\ Z=\sum_{i}^{N}w_{i}^{0}\exp\left(\frac{-V_{i}^{bias,opt}}{k_{B}T}\right) \end{array}$$

Even though the shape of the optimal biasing potential is unknown, the sums of harmonic potentials have emerged as an appropriate approximation [36]. We therefore assume that $V^{bias}$ (Equation (9)) is linearly dependent on the squared deviation between the calculated and experimental observables. One constant $k_{i}$ is assigned to each observable $O_{i}$ and can be interpreted as the influence of the observable on the biasing potential.

(9) $$V^{bias}\left(k,t\right)=\sum_{i}^{M}\frac{1}{2}k_{i}*{\left(\frac{O_{i}^{exp}-O_{i}^{calc}\left(t\right)}{\sigma_{i}}\right)}^{2}$$

where k is the force-constant-like vector with elements $k_{i}$.

Combining Equations (8) and (9) shows the relation between the vector k and weight vector w (Equation (10)). A large $k_{i}$ implies that conformations with a disagreement between the simulated and experimental results have weights close to zero. Therefore, $k_{i}$ can be interpreted as the value, showing how sensitive the reweighting is to the deviation of an observable.

(10) $$w_{t}\left(k\right)=\frac{w_{t}^{0}}{Z}\exp\left(\frac{-V_{t}^{bias}\left(k\right)}{k_{B}T}\right)=\frac{w_{t}^{0}}{Z}\exp\left(\frac{-\sum_{i}^{M}0.5k_{i}{\left(\frac{O_{i}^{exp}-O_{i,t}^{calc}}{\sigma_{i}}\right)}^{2}}{k_{B}T}\right)$$

### 2.1. Optimizing the k-Vector

Equation (10) describes the relation between the ensemble weights, the biasing potential, and the k-vector. This relationship is essential to calculate the weights $w^{opt}$ (size *N*) from the optimized k-vector (size *M*). It is now necessary to optimize the k-vector such that the optimized weights lead to an ensemble for which the ensemble averages of the calculated observables show an improved agreement with their experimental counterparts. In our recent review [41], we discussed ensemble reweighting using Bayes’ theorem in detail, which allows for us to calculate the conditional probability of events. Equation (11) shows the theorem in its simplified form. P(w|data) is the posterior probability of the weights w given these data. The maximization of this probability leads to the optimal weights for a given set of experimental data. P(data|w) is the conditional probability that the data can be reproduced given a set of weights w and $P_{0}\left(w\right)$ is the estimated probability of being correct before any data are observed.

(11) $$P\left(w|data\right)\propto P\left(data|w\right)*P_{0}\left(w\right)$$

The conditional probability and the prior probability can be modeled with the $X^{2}$ error (Equation (13)) and the Kullback–Leibler divergence (Equation (16), [42]), respectively, leading to Equation (12). A scaling factor theta is added to balance the agreement with the experiment and the divergence from the original weights.

(12) $$P\left(w|data\right)\propto \exp\left(-X^{2}\right)*\exp\left(-\theta D_{KL}\right)$$

(13) $$X^{2}\left(w\right)=\frac{1}{M}\sum_{i}^{M}{\left(\frac{O_{i}^{exp}-\sum_{t}^{N}w_{t}O_{i}^{calc}\left(t\right)}{\sigma_{i}}\right)}^{2}$$

Note that the $X^{2}$ error depends explicitly on the weighted ensemble averages of the calculated observables. Bayesian Ensemble Refinement typically uses a cost function that can be derived by rearranging Equation (12).

(14) $$\min_{w}\;\cos t\left(w\right)=\theta *D_{KL}\left(w\right)+X^{2}\left(w\right)$$

The cost function (Equation (14)) is then minimized to find the appropriate vector $w^{opt}$ that refines the ensemble. To overcome the necessity of directly minimizing *w*, we introduce the relationship between the typically much smaller k-vector and the weights w (Equation (10)), as discussed in the previous chapter. We now minimize the cost function, which is dependent on the small k-vector instead of the typically much larger number of weights.

(15) $$\min_{k}\;\cos t\left(k\right)=\theta *D_{KL}\left(k\right)+X^{2}\left(k\right)$$

#### Umbrella Refinement and the Maximum Entropy Principle

While Bayes’ theorem does not always seem intuitive, Equation (15) can also be justified when starting from the maximum entropy principle.

Jaynes [43,44] formalized the maximum entropy method to find a probability distribution that is consistent with known constraints, e.g., the agreement of ensemble averages with experimental observations. By maximizing entropy, the method yields the least-biased estimate given the available solutions and constraints [45,46]. Entropy-maximizing ensemble refinement [30,32,33,47,48,49,50,51,52,53,54] is an established group of methods that build upon this principle to optimize the statistical weights of a conformational ensemble while trying to leverage the initial information obtained from molecular dynamics simulations and balance it with the experimental data.

The relative entropy, or Kullback–Leibler divergence (KL divergence, $D_{KL}$, Equation (16) or (17)), measures the difference between two probability distributions P(x) and $Q_{v}\left(x\right)$. In our method, P(x) is represented by the constant initial weights $w^{0}$ while $Q_{v}\left(x\right)$ is represented by the weights w, which are optimized. $D_{KL}$ quantifies the amount of information lost when using distribution $Q_{v}\left(x\right)$ as a model to approximate distribution P(x). If minimized subject to a set of constraints, the distribution $Q_{v}\left(x\right)$ can be assumed to be the distribution that requires minimal additional information [55]. As the difference between the initial and optimized ensembles is quantified by the KL-divergence (Equations (16) or (17)), it is used in Equation (14) to regularize the strength of optimization during the reweighting of the ensemble.

Due to the non-symmetry of the KL divergence, the direction of the comparison is of substantial importance. The forward direction is called mode-covering, while the reverse formulation is called mode-seeking. In the Supplementary Materials, we present an example that demonstrates the different behaviors of a KL divergence-guided minimization, which are dependent on the direction of comparison. The different minimization behaviors are discussed in detail in our recent review [41].

(16) $$D_{KL}\left(P|\right|Q_{v}{)}_{forward}=\sum_{x}P\left(x\right)*\ln\frac{P(x)}{Q_{v}\left(x\right)}$$

(17) $$D_{KL}(Q_{v}{\left||P\right)}_{reverse}=\sum_{x}Q_{v}\left(x\right)*\ln\frac{Q_{v}\left(x\right)}{P(x)}$$

Following the idea of maximum entropy, a conformational ensemble close to the initial distribution should be found, making the KL divergence the ideal metric to quantify this derivation. Both the forward and the reversed direction of the KL divergence can be used in ensemble refinement. To avoid the trivial solution of no reweighting at all, it is also necessary to constrain the optimization in regard to the deviation between the experiment and simulation. The constraint design now offers two options:

1. Per-Observable Constraints
  The classical solution of the maximum entropy method, leveraging Lagrange multipliers, minimizes the KL divergence under the condition of multiple per-observable constraints. If the reversed KL divergence is used, this approach offers a fast solution to the minimization problem, incentivizing the use of this direction if per-observable constraints are used.
2. The Global $X^{2}$ Constraint
  Alternatively, a constraint could be set not on the individual observables but on the $X^{2}$ value, a metric that measures an average-like deviation between the experiment and simulation for all tracked observables. This metric allows for errors to compensate each other and tolerates some observables deviating as long as the majority are compliant.

Combining both arguments, an optimal set of weights minimizes both the $X^{2}$ objective and the KL-divergence simultaneously. As optimization under constraints is typically computationally expensive, the unconstrained and simultaneous minimization of the two terms is preferred. To create such a loss function, both terms that should be minimized are added up, and balanced by a hyper parameter (theta, θ) that balances both terms and defines the strength of refinement. This leads directly to Equation (15), which was previously reasoned using Bayes’ theorem.

While the methodology of the Umbrella Refinement of Ensembles can be used with both the forward and reversed direction of the KL divergence, we chose the forward direction for all calculations in this work to leverage the mode-covering behavior.

### 2.2. Estimation of the Hyper-Parameter Theta

The hyper-parameter theta (θ) sets the strength of the optimization and can be freely tuned. A high value of θ gives importance to the KL divergence, ensuring that the reweighted ensemble stays close to the initial reference. On the other hand, a small θ value reduces the importance of the KL divergence, letting the cost function be guided by the $X^{2}$ term. A well-chosen θ avoids overfitting the data while allowing for sufficient reweighting.

Bottaro et al. [56] describe a five-fold cross-validation to estimate the optimal value of theta. The data are split into training and validation sets. For different values of theta, the optimized weights w are calculated using the training data only. These weights are then used to calculate $X^{2}$ using only the data from the validation set. As validation score, $X^{2}/X_{init}^{2}$, is calculated where $X_{init}^{2}$ is computed using the initial weights $w^{0}$.

**Figure 2:**
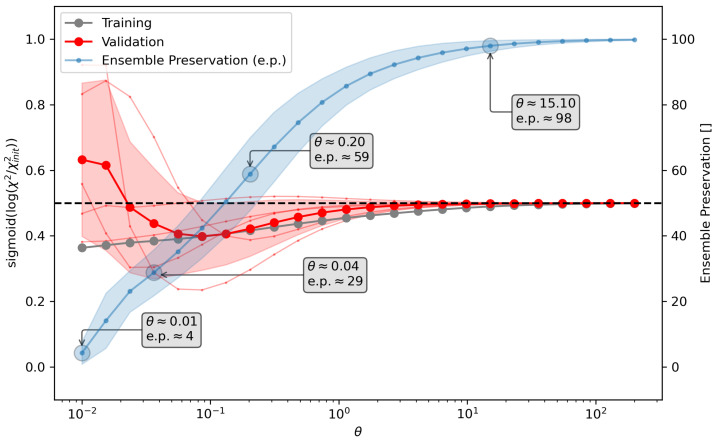
Cross-validation using a five-fold testing/validation split was used to determine a proper value for the hyper parameter theta (θ). The *x*-axis shows θ while the left *y*-axis shows the validation score ($sigmoid(log\left(X^{2}/X_{init}^{2}\right))$). Gray represents the validation score when compared with the training data while red represents the score when compared with the validation data. Thin red lines indicate five individual splits in the data; the thick curve and shaded area indicate the average and the standard deviation, respectively. The ensemble preservation (right *y*-axis) indicates the share of the initial ensemble that remains after reweighting. The blue solid line represents the average of the ensemble preservation calculated from the training data and the shaded area represents the standard deviation. Four choices of theta are marked, representing overfitting (the leftmost mark), two reasonable choices (the two center marks), and underfitting (the rightmost mark). The graphic shows the cross-validation of the alanine–alanine zwitterion reweighting performed in Section 3 using uniform initial weights and the URE method. Cross-validation plots of the other sets of initial weights that were tested can be found in the Supplementary Material.

In this work, we apply a modified variant of this cross-validation scheme. To obtain the validation score, we calculate $sigmoid(log\left(X^{2}/X_{init}^{2}\right))$, which limits the value range to a number between zero and one. If a set of weights improves the agreement between the simulation and experiment not only in regard to the fitted observables, but also in regard to previously unknown ones, a validation score lower than 0.5 is calculated. On the other hand, a higher validation score must be interpreted as a set of weights that worsens the agreement between the simulation and experiment for the validation data, indicating possible overfitting of the data. Typically, a clear minimum with little noise, as shown in Figure 2, indicates a very well-suited reweighting of the conformational ensemble. The θ value of this minimum is a proper choice to perform a reweighting of the entire dataset. If a distinct minimum cannot be found, θ values that lead to increased validation scores should be avoided and the results of the optimization need to be checked carefully.

If the cross-validation score does not help to determine θ, we additionally introduce the ensemble preservation metric (e.p.). Overfitting in the context of conformational ensemble refinement typically leads to the selection of a very low number of conformations with significant weights, while the vast majority of conformations are assigned weights of zero. Overfitting may numerically improve the $X^{2}$ metric but creates an ensemble that is no longer plausible as a description of the biophysical nature of a molecule. The ensemble preservation serves as an available indicator of this distortion. The unchanged ensemble has a preservation of 100, with more distortion leading to a lower metric. A more detailed description of the methodology can be found in the Supplementary Material, ESI Section 1.1.

## 3. Validation

### 3.1. Methods

To investigate the behavior of the URE method, as described in Section 2, a reference implementation to reweight ensembles against chemical shifts, J3 couplings, and NOE-derived bounds on intermolecular distances was implemented. The code is available, as indicated in the data availability statement. The input format of the data, as well as the preconditioning, is designed to work similarly to the established method of Bottaro et al. [56].

Two input files are required—one containing experimental data and one containing calculated observables for each conformation of a simulation. In addition to the experimental observables, the expected uncertainty σ is required to indicate the confidence in the input data. As it is possible to provide data from different experimental methods, preconditioning of the data is required, which is carried out automatically by the software. Interatomic distances that are to be compared to NOE-derived bounds are linearized by raising them to the power of minus six ($r^{-6}$), which allows these observables to be treated by linear averaging during the optimization. The thus-obtained vector of the experimental data $O^{\exp}$ and matrix of simulated data $O^{\mathrm{calc}}\left(t\right)$ is then used to minimize the representative loss function to calculate the k-vector.

In addition to other explicitly mentioned software, the open source packages JAX 0.4 [57], MDAnalysis 2.9 [58,59], SciPy 1.11 [60], NumPy 1.26 [61], Pandas 2.2 [62,63], and Matplotlib 3.8 [64] were used in this study.

#### 3.1.1. The Alanine–Alanine Zwitterion

The alanine–alanine zwitterion, as described by Bou$\overset{˘}{r}$ et al. [65], was chosen as the test system. The small system size and the low number of degrees of freedom allow for the complete sampling of the conformational space. The chemical identity of the dipeptide, the system temperature, and the omega angle were preserved during the entire study, thus reducing the degrees of freedom to only the phi and psi angles of the molecule. The phi- and psi angles of the system were discretized into bins of 10 degrees, creating an ensemble of 36 × 36 structures that describe all relevant conformations of the dialanine.

For each of the resulting 1296 conformers, the free-energy surface was calculated using three established energy functions (GROMOS 54a8bb [66] within GROMOS [67,68], Amber ff14SB [69] within OpenMM [70], and PM7 [71] within MOPAC [72]). After calculating the free energy for each of the resulting 1296 conformers, a probability of occurrence $w_{t}$ was calculated using the Boltzmann distribution. In addition, an equipotential system was introduced to evaluate the performance of reweighting if no prior information is available; hence, the initial weights were equal for all 1296 conformers. Four reweighting attempts were performed, each using the same initial ensemble obtained from the geometry-optimized conformations by MOPAC using the PM7 energy function. The only difference between the reweighing experiments were the four different estimates of the initial weights.

GROMOS 54A8bb

The simulation of the zwitterion was set up using the GROMOS 54a8bb force-field with explicit SPC [73] water (1023 molecules) and the Nosé–Hoover Chains [74,75] thermostat. After equilibrating to 300 K, a 20 ns long local-elevation [76] simulation was performed to build up an appropriate umbrella potential for a subsequent production run with constant potential. The omega dihedral of the zwitterion was restrained using a harmonic restraint with a force constant of 0.0381 kJ/mol/degree^2^ to maintain its transconfiguration. Both the phi and the psi dihedral were accelerated using one two-dimensional periodic umbrella potential (LEUSBIAS, with a CLES value of 0.005). The production run was another 20 ns, which allowed the conformational space to be sampled and the (binner) free-energy landscape to be calculated.

Amber ff14SB

Similarly, the free energy surface of the zwitterion was calculated using the metadynamics [77] method of OpenMM. Amber ff14SB with the TIP3P [78] water model was chosen as the force-field. The omega angle was restrained using a custom torsion force with a force constant of 125 kJ/mol. Both the phi and psi angles were accelerated by adding Gaussian bumps with a height of 1 kJ/mol using a biasFactor of 4.0 every 50 steps. The free energy profile was calculated using the Langevin integrator with an integration interval of 2 fs [79] during the 10 ns long simulation at 300 K.

MOPAC PM7

The MOPAC 22.1 software was used to geometry-optimize the entire conformational ensemble using the PM7 hamiltonian. The omega dihedral was constrained to 180 degrees and both the phi and psi dihedral angles were adjusted to the appropriate value for the given conformer. After the constraint geometry optimization of the ensemble, the thermodynamic properties were calculated. For the calculation of the free-energy surface, a temperature of 300 K was assumed.

Equipotential

To test the behavior of the reweighting method if no prior information is provided, a flat free-energy surface with equipotential initial weights for all conformations was chosen.

Observables

The observables of each conformer of the ensemble were calculated using the optimized geometries of the MOPAC ensemble. To calculate chemical shifts, the software UCBShiftX version 1 of Li et al. [80] was selected. From the 12 calculated chemical shifts, 10 were considered suitable for the reweighting. The remaining two chemical shifts from the amine end group were disregarded as non-representative. An error estimate was obtained from UCBShift’s original publication [80]. The total error when combining both the simulated and experimental error was assumed to be twice the estimated error of the simulation.

The J3 couplings were independently calculated using the Karplus equation [81,82] with three different parameter sets [83,84,85]. Using the three predictions, an average and a standard deviation of each coupling were calculated. The average J3 couplings were used to reweight the ensemble, while the doubled standard deviation was assumed to approximate the total error from both the experiment and the simulation.

Experimental values were obtained from Bou$\overset{˘}{r}$ et al. [65,86], where ten chemical shifts and two J3 couplings were selected as the reweighting targets.

Reweighting

A θ estimation was performed using five-fold cross-validation. Due to the small number of observables, a test/validation split of 75/25 was chosen to improve stability. With the use of cross-validation, we were able to find a suggested value for θ with three different sets of initial weights but failed with the weights obtained from the Amber ff14SB simulation. The θ recommendations from the other three cross-validations (where the minima of the cross-validation curves $\theta_{curve-min}$ are 0.483, 0.183 and 0.070) were used to choose one mutual value to allow for a comparison between the reweighted ensembles. It was estimated that a theta of 0.2 was reasonable, and this was thus chosen for all four reweighting operations.

#### 3.1.2. Comparison to Bottaro et al. [52]

To verify the behavior of the Umbrella Refinement of Ensembles method, the reweighting of the dialanine zwitterion was performed using not only our URE method, but also the established Bayesian/Maximum Entropy (BME) approach, as introduced and implemented by Bottaro et al. [52]. The code of the BME method was downloaded from the source linked in their publication. Using the cross-validation from Figure 2, four different values for θ were selected, one underfitting, one overfitting, and two in the recommended θ-range. The calculated ensemble preservation (e.p.) value was calculated for each of the four reweighting attempts. The dialanine zwitterion system with equipotential initial weights was reweighted using both the URE and the BME method. With the BME method, the value for θ was chosen such that the ensemble preservation of the four reweightings was comparable to the reweightings with our URE method.

#### 3.1.3. Chignolin

Chignolin is a small peptide designed to feature fast folding and was first described by Honda et al. [87] Its initial structure was obtained from the PDB database (entry 1UAO). Initially, a 1001 ns long denaturation simulation was performed at 500 K. From this trajectory, five different conformers with an RMSD of at least 7 Å compared to the reference structure and with a confirmed trans conformation of residue 4 (proline) were extracted and used as the initial conformation of five independent folding simulations.

A total of five folding simulations were calculated using the Amber ff14SB force-field with the OpenMM 8 software. The polypeptide was solvated in 4000 TIP3P water molecules and $Na^{+}Cl^{-}$ ions, creating an ionic concentration of 60 mM. The protonation was set to represent pH 5.5. The simulation was performed using nonbonded PME interactions with a 2 fs integration interval using the LangevinMiddle integrator with a reference temperature of 277 K and a barostat set to 1.0 atm. Each of the five replicas was simulated for 10 μs, each creating a npT ensemble with 1 million configurations written out at regular intervals. Due to the correlated nature of the MD trajectories, an equally sampled, sparse trajectory containing 1% of the configurations was generated for further analysis.

Earlier investigations [88,89,90] demonstrate that chignolin can be observed in one of three states: native fold, misfolded and unfolded. Multiple methods to classify single conformers into one of the three states have been proposed; in this study, we use a classification based on Kührová et al. [88], which separates native and misfolded conformations based on whether Gly-7 adopts the $\beta_{PR}$ or $\alpha_{L}$ conformation. A conformation is assumed to be unfolded if the radius of gyration is bigger than 6.3 Å and the RMSD with respect to the reference structure is bigger than 3.0 Å.

The experimental observables used to reweight the ensemble were obtained from BMRB Entry 5694 (chemical shifts, J3 couplings) and RCSB entry 1UAO (NOEs). The experimental data, measured by the original authors Honda et al., [87] were obtained at 277 K and a pH of 5.5. From the 57 initially obtained chemical shifts, 14 were selected for the reweighting. Chemical shifts with an ambiguous assignment and those from terminal residues and sidechains were disregarded. The chemical shifts from the simulation and the associated expected uncertainty were calculated as described for the dialanine zwitterion. Of 16 J3 couplings, 8 were non-ambiguous and chosen for the reweighting. The J3 coupling constants of the simulation were also calculated in the same way as described for the dialanine zwitterion, but the expected uncertainty was assumed to be the same as that reported in the literature. Out of the substantial number of experimentally reported NOEs, ambiguous restraints were excluded, leaving 32 NOE upper bounds to be included in the reweighting. The simulated NOE-derived interatomic distances were calculated using the SPyCi-PDB software version 0.4.3 [91] and the associated uncertainty of these distances was postulated to be 0.5 Å.

The reweighting was conducted using the Umbrella Refinement of Ensembles method. After taking the cross-validation and ensemble preservation score into account, θ was set to 0.05. The reweighting of all five ensembles was performed using the same θ value.

### 3.2. Results

#### 3.2.1. The Alanine–Alanine Zwitterion

**Figure 3:**
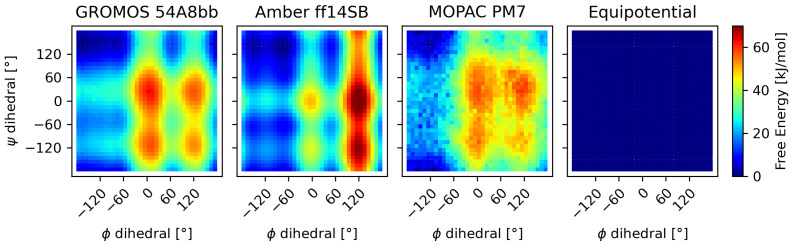
The alanine–alanine zwitterion allows for the free-energy surface of the molecule to be sampled due to its small number of degrees of freedom. All three methods show one major minimum and multiple weaker relative minima. To evaluate the reweighting of the system without prior information using uniform initial weights, the equipotential surface was added to the comparison.

**Figure 4:**
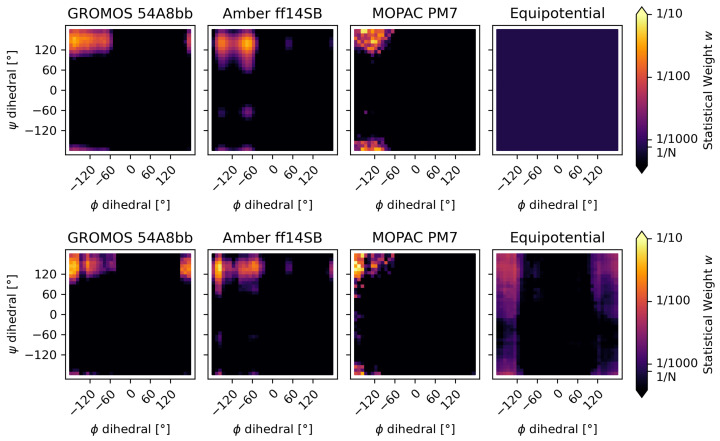
Using Boltzmann’s distribution, the initial weights $w^{0}$ (top row; brighter indicates a higher weight) were calculated from the free energy estimates from the three different energy functions—GROMOS 54A8bb, Amber ff14SB and MOPAC PM7—plus the equipotential surface. The top row, therefore, shows the probability of occupation (statistical weights) before the reweighting. The three systems with prior weights show that almost the whole population was found in the major minimum. The lower row shows the same systems after reweighting using the URE method. While the systems with non-uniform prior weights show only subtle changes, the reweighted equipotential system now shows a major population close to the major minimum of the expected potential energy surface and a second, weaker, population at its inverted phi angle.

The free-energy energy surface of the alanine–alanine zwitterion shows a wide minimum, which can be seen in the β-sheet region of the plots in Figure 3. The existence and approximate location of this minimum were confirmed independently by all three methods employed to calculate the free-energy surface, as well as by other theoretical and experimental studies [65]. As expected, the calculated probability of occurrence (weights, Figure 4) for the conformers within this minimum sum up to nearly one, leaving all other areas of the potential energy surface practically unpopulated.

After reweighting the ensembles, each of them using their prior weights, the now-refined weights $w^{\mathrm{opt}}$ changed slightly within the minima but did not change the general assessment of the system. Reweighting the ensemble with uniformly distributed initial weights (equipotential system) yielded an ensemble with a significant population in the expected β-sheet region, even through a minor population is also predicted to exist, with an inverted phi angle.

**Figure 5:**
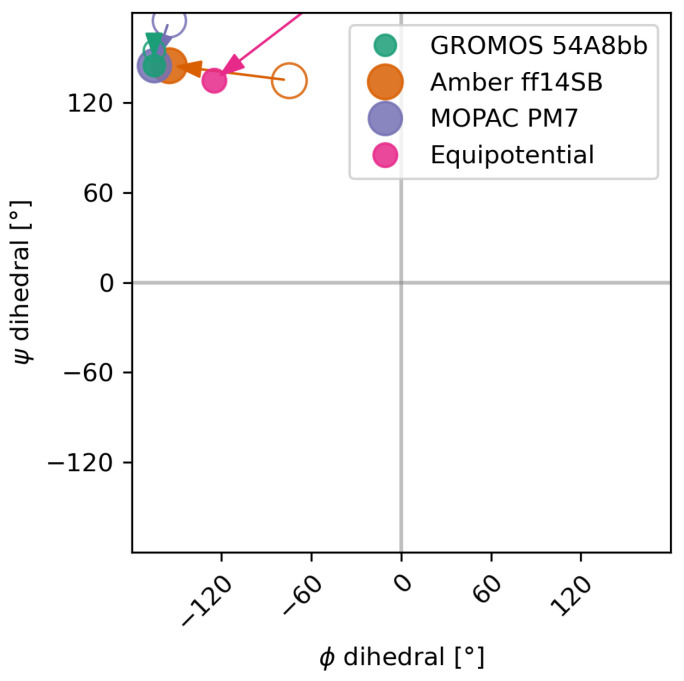
The most prominent conformation of the ensemble changes due to the reweighting in all four cases. It can be observed that the estimated minimum of the free energy surface converges together after the reweighting. Circles indicate the position of the minimum before the reweighting; solid dots indicate the position after the reweighting. The equipotential case has no initial minimum but an optimized one.

To assess the quality of the ensemble reweighting, we checked whether the predicted highest-weight conformation of each method converged after reweighing to one mutual solution, independently of the initial weights. Figure 5 shows the shift in the conformation with maximal statistical weight due to the ensemble optimization. It can be seen that the shift in the systems calculated using GROMOS 54A8bb and MOPAC PM7 is minor, shifting only to a bin in close proximity. The shift in the initial weights according to Amber ff14SB is more significant as the reweighting changed the most populated minimum of the system. Before the reweighting, the weights calculated by the Amber force-field indicated two populated minima at phi dihedral angles of around −75 (major) and −155 (minor) degrees, respectively. The reweighting inverted that population, putting most of the weight at around −155 degrees while depopulating most of the −75 degree minimum. Using uniform initial weights, the optimized ensemble is substantially more diverse than the ensembles using non-uniform initial weights, as is to be expected from maximum entropy methods when taking prior information into account. Table 1 shows the reported metrics of the optimization with the URE method.

#### 3.2.2. Comparison to Bottaro et al. [52]

**Figure 6:**
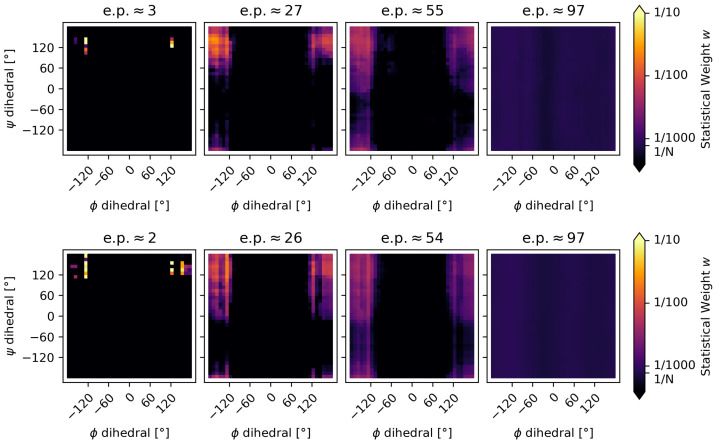
The alanine–alanine zwitterion was reweighted using the URE method (**top**) and the BME method of Bottaro et al. (**bottom**). The strength of the reweighting increases from left to right. The leftmost subplots demonstrate the effect of overfitting, where only some conformers from the initial ensemble are chosen, resulting in a strongly distorted ensemble characterized by a low ensemble preservation factor (e.p.). On the other hand, the rightmost subplots demonstrate underfitting from a high θ value, leading to only a negligible change in the ensemble, which does not show the relevant β-sheet region. The center subplots show a reasonable level of reweighting.

The comparison of our URE method with the established Bayesian/Maximum Entropy (BME) approach from Bottaro et al. [52] confirms the validity of the method. Using the same initial weights and the same initial ensemble and experimental data, the results of the reweighting are remarkably similar. Figure 6 and Table 2 shows a comparison of the results, where a group of conformations consistent with the β-sheet region is promoted with both reweighting methods. The URE reweighting shows a particularly smooth probability surface, where the low level of noise leads to a focused new ensemble with most conformers of the relevant β-sheet region being well represented.

#### 3.2.3. Chignolin

**Figure 7:**
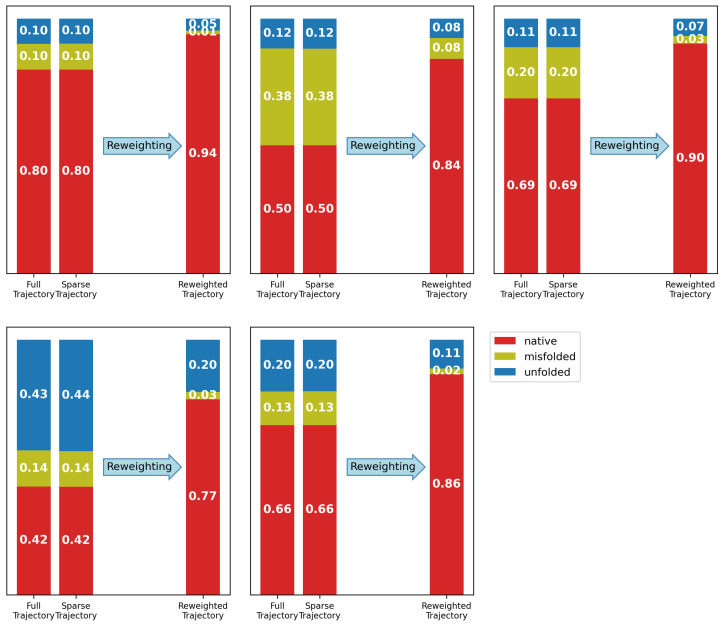
The five replicas of the MD simulation of chignolin can be used to estimate the shares of chignolin’s major conformations. Both the full and sparse trajectories show the same share of conformations, thus confirming that the sparsification does not substantially alter the ensemble. Reweighting was performed on the sparse trajectory, increasing the share of the natively folded conformation, while the misfolded conformation is substantially reduced.

Both dense and sparse trajectories of each MD replica were analyzed regarding their major conformations (native fold, misfolded, and unfolded). It can be seen (Figure 7) that the sparsification of the data at regular intervals did not influence the conformational composition of the trajectories. Four of the five replicas show relatively similar initial weights in the unfolded state, while one (replica 4) maintains a higher-than-expected share of unfolded conformations. Replica 2, on the other hand, shows a comparably frequent occurrence of misfolded structures. There were multiple observations of folding, unfolding, and refolding events (ESI Figure S9 and Figure S10). Interestingly, the transition between the two folded conformations often includes an unfolded transition state with a very short lifetime. Even with simulation length of 10 μs, the number of transitions between conformational states is not sufficient for the same initial weights to be observed for the three conformational states in the five replicas.

The individual reweighting of each of the five trajectories (Figure 7) shows an increase in the natively folded conformation, while the unfolded conformations maintain a share of around 10%. The experimental data from Honda et al. [87] estimate the share of folded chignolin at 277 K to be around 80%. Therefore, the share of the folded conformation is likely to be slightly overestimated after the reweighting. The ensemble preservation remains between 35 and 42, confirming that the ensemble distortion due to the reweighting remained limited. While the reweighting did not remove all differences between the initial weights of the replicas, the five reweighted ensembles are more in agreement than the initial ones.

## 4. Conclusions

We introduced the forward-formulated Umbrella Refinement of Ensembles (URE) method in the context of maximum entropy ensemble refinement. This combines the advantage of optimizing a lower number of parameters k with the mode-covering behavior of the forward formulated KL-divergence, a characteristic that is typically not available with common Lagrange multiplier-based ensemble optimizers. Additionally, the introduction of the k-vector as a parameter directly derived from Umbrella Sampling provides an opportunity for a physical interpretation of the results.

Umbrella Refinement demonstrated the ability to yield ensembles compatible with the experimental data. The full sampling of the relevant conformational space of the dialanine zwitterion allowed us to characterize and verify the correct behavior of the method. The initial weights obtained with the Amber ff14SB force-field showed two separated local minima in close proximity, where the population at higher phi-dihedral angles is preferred. Reweighting inverted this preference to favor the population at lower phi-dihedral angles, as was seen with the GROMOS 54A8bb prior. Using uniform initial weights, the behavior of the URE method was compared to the established BME method, confirming the validity of the results and the high degree of smoothness of the calculated probability surface calculated by the URE method. Finally, both methods show similar over- and underfitting characteristics if inappropriate θ-values are used.

Five replicas of chignolin simulations confirmed the peptides’ ability to fold, unfold, and refold at a sub-microsecond timescale. The simulated period of 10 μs was not long enough to converge the expected share of major conformations in the trajectories. Reweighting using the URE method led the results to converge further, demonstrating the ability to draw conclusions about geometrical properties from these reweighted ensembles. It was shown that an ensemble dominated by the natively folded conformer can be expected in the case of chignolin. The calculated share of folded conformations was slightly higher than that previously reported in the literature.

While the validation of the URE method showed promising results, we emphasize that reweighting methods require both simulated and experimental data and prior weights to be well-curated. The results from the dialanine zwitterion suggest that reweighing using no prior weights may be preferable to using low-confidence initial weights. However, when considering larger molecules, the relation between the degrees of freedom and the available experimental datapoints typically becomes unfavourable, such that the number of degrees of freedom increases faster than the number of datapoints. Calculating the optimal weights of the ensemble remains an mathematically underdetermined problem in view of the number of experimental datapoints, such that any reweighting scheme can only be expected to adjust a reasonable estimate of initial weights. If poorly curated data are used during the process of reweighting, this may lead to misleading findings that are difficult to spot and incorrect findings. In summary, however, it can be stated that reweighting works well if used carefully, with well-curated data. Maximum entropy methods provide a solid theoretical foundation and possess promising properties to integrate simulated and experimental data, allowing for new and exciting insights into molecular behavior.

## Figures and Tables

**Table 1:** The GROMOS 54A8bb, Amber ff14SB, and MOPAC PM7 energy functions were used to calculate the free-energy surface for the zwitterion. The initial weights were calculated from the corresponding Boltzmann distribution. The value $\theta_{curve-min}$ represents the minimum of the validation–value curve obtained from the five-fold cross-validation; $\theta_{applied}$ is the value used to reweight the ensemble. The value $X_{initial}^{2}$ represents the agreement between simulated and experimental observables before the reweighting, and $X_{opt}^{2}$ represents the agreement afterwards. Smaller values for $X^{2}$ represent better agreement. The minima of the free-energy surface were calculated using a grid with bins of 10 degrees, thus limiting the precision of the estimation to this threshold. The constant value of 0.2 for $\theta_{applied}$ was chosen to allow for a comparison between the results. The cross-validation using weights obtained with Amber ff14SB failed to yield a minimum value; therefore, no theta can be recommended from the cross-validation.

Initial Weights	$\theta_{\mathrm{curve}-\min}$	$\theta_{\mathrm{applied}}$	$X_{\mathrm{initial}}^{2}$	$X_{\mathrm{opt}}^{2}$	Min_initial_ [°]	Min_optimized_ [°]
54A8bb (GROMOS)	0.483	0.200	1.307	0.800	(−165, 155)	(−165, 145)
Amber ff14SB (OpenMM)	–	0.200	1.355	0.961	(−75, 135)	(−155, 145)
PM7 (MOPAC)	0.183	0.200	2.159	0.904	(−155, 175)	(−165, 145)
Equipotential	0.070	0.200	1.514	0.931	–	(−125, 135)

**Table 2:** The quantification of the comparison between the URE and BME methods confirms the results visualized in Figure 6. Both methods yield very similar results given the same initial ensemble, weights, and experimental data. Due to the different algorithmic designs, the values of θ are not transferable between the methods. Once θ is adjusted to reweight at the same strength, both the ensemble preservation (e.p.) and the $X^{2}$ deviation between the experimental data and simulated expectation value show comparable values.

	URE Method			BME Method		
**Reweighting Strength**	$\theta_{\mathrm{applied}}$	$X_{\mathrm{opt}}^{2}$	**e.p.**	$\theta_{\mathrm{applied}}$	$X_{\mathrm{opt}}^{2}$	**e.p.**
underfitting	15.10	1.42	97	111.38	1.42	97
good	0.20	0.93	55	1.30	0.92	54
good	0.04	0.80	27	0.40	0.78	26
overfitting	0.01	0.72	3	0.02	0.71	2

## Data Availability

Scripts and input files to rerun the simulations and data analysis, as well as the reference implementation of the URE method, can be downloaded from https://doi.org/10.5281/zenodo.14733012 (accessed on 26 May 2025).

## Supplementary Materials

Supporting information can be downloaded at: https://www.mdpi.com/article/10.3390/molecules30112449/s1. Refs. [92,93,94] are cited in the Supplementary Materials,.
