# Supplementary material for: Umbrella Refinement of Ensembles—An Alternative View of Ensemble Optimization
Source: Molecules. 2025 Jun 3;30(11):2449. doi: 10.3390/molecules30112449 (PMC12156320; doi:10.3390/molecules30112449)
Supplement: Supplementary file 1 [file molecules-30-02449-s001.zip › molecules-3633242-supplementary.pdf]

Umbrella Refinement of Ensembles - An Alternative View on Ensemble Optimization

Supplementary Material

Johannes STÖCKELMAIER[1], Tümay CAPRAZ[2] and Chris OOSTENBRINK[1,3]
1. Institute of Molecular Modeling and Simulation (MMS)
BOKU University, Vienna
2. European Molecular Biology Laboratory (EMBL), Heidelberg
3. Christian Doppler Laboratory Molecular Informatics in the Biosciences
BOKU University, Vienna

June 2, 2025

1 Ensemble Preservation

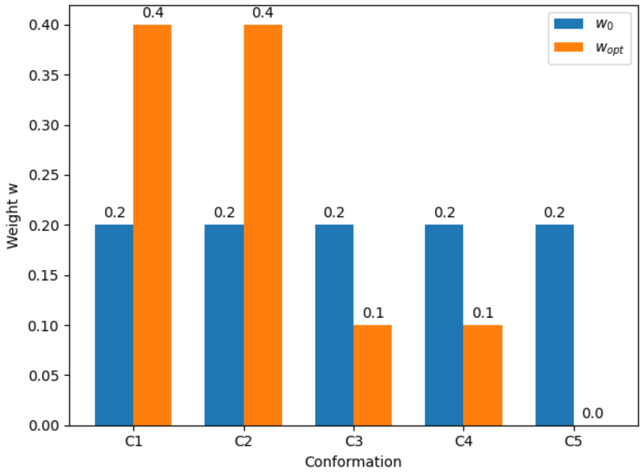

Figure S1: To determine a reasonable value for theta, the metric of ensemble preservation is introduced. Using small values for theta it may happen that only few conformations remain in the ensemble, thus leading to a significant distortion of the initial ensemble. The ensemble preservation serves as an approachable indicator for this distortion. The unchanged ensemble equals a preservation of 100. In the example above the ensemble preservation can be calculated (compare eq. 1) as $\frac{100}{5} * (1 + 1 + 0.5 + 0.5 + 0) = 60$.

$$\text{ensemble preservation} = \frac{100}{N} * \sum_{t}^{N} \begin{cases} w_t^{opt}/w_t^0 & \text{if } w_t^{opt} < w_t^0 \\ 1 & \text{if } w_t^{opt} \geq w_t^0 \end{cases} \tag{1}$$

2 The Direction of the Kullback-Leibler Divergence - An Example

The Kullback-Leibler (KL) divergence allows for two distinct directions in comparing probability distributions. This difference becomes particularly apparent when optimizing systems with a limited number of parameters. A frequently shown example, illustrated in Figure S2, demonstrates fitting a single Gaussian distribution (with the two parameters μ and σ) to a bimodal reference distribution. The parameters are determined by minimizing the KL divergence between the two distributions. Optimizations employing the forward KL divergence (Equation 2) are termed mode-covering (inclusive), while optimizations using the reversed KL divergence (Equation 3) are referred to as mode-seeking (exclusive).[1–3]

$$D_{KL}(P||Q_v)_{forward} = \sum_x P(x) * ln \frac{P(x)}{Q_v(x)} \tag{2}$$

$$D_{KL}(Q_v||P)_{reverse} = \sum_x Q_v(x) * ln \frac{Q_v(x)}{P(x)} \tag{3}$$

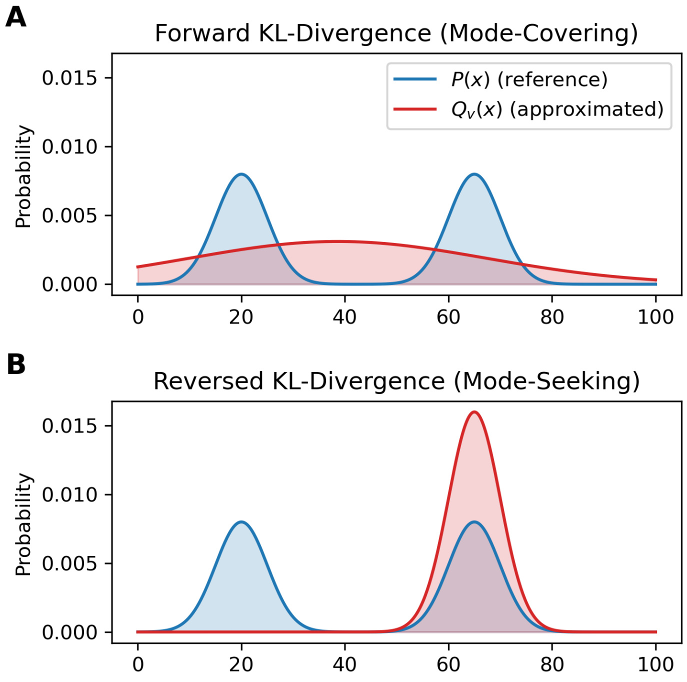

Figure S2: Illustration of the differing behavior of the forward and reversed Kullback-Leibler (KL) divergence during optimization. The reference distribution, $P(x)$ (blue) is a bimodal sum of two Gaussian functions ($\mu_1 = 20$; $\mu_2 = 65$; $\sigma_{1,2} = 5$) normalized to one. The optimized distribution, $Q_v(x)$ (red) is a single Gaussian with optimized parameters μ and σ, found by minimizing the KL divergence between $P(x)$ and $Q_v(x)$.

3 The alanine-alanine zwitterion

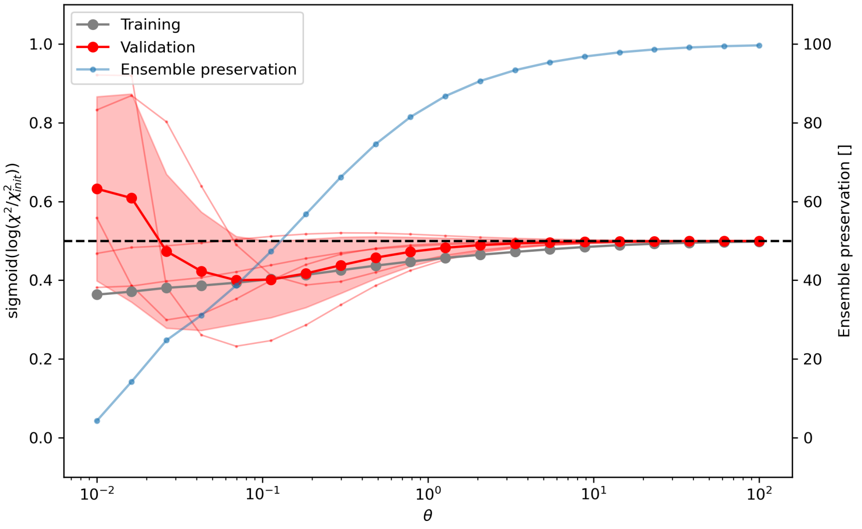

Figure S3: Cross validation of the Zwitterion reweighting using uniform prior weights from the equipotential system. The x-axis shows the chosen value of theta while the y-axis shows the error between experiment and simulation. Gray represents the error against the training data while red represents the error against the validation data. For the final reweighting the value of theta which represents the minimum of $\mathcal{X}^2$ against the validation set is recommended if the ensemble preservation (blue) is sufficient.

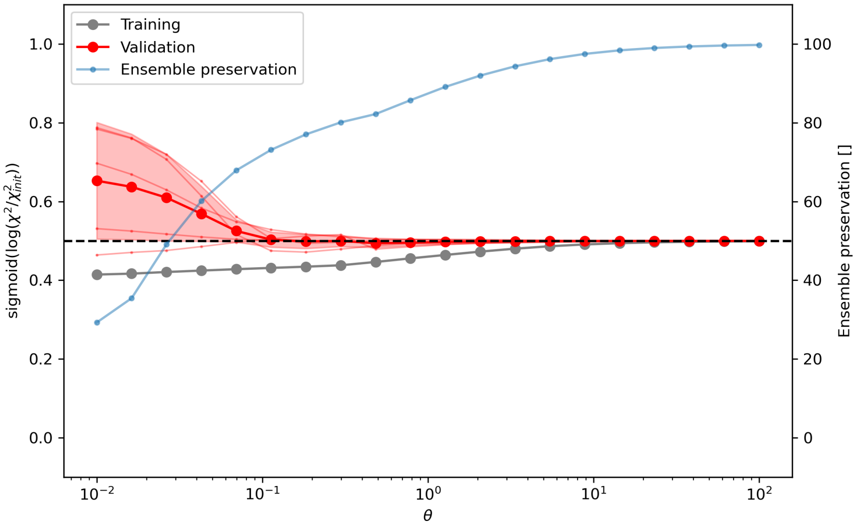

Figure S4: Cross validation of the Zwitterion reweighting using prior weights from calculations with GROMOS 54A8bb. The x-axis shows the chosen value of theta while the y-axis shows the error between experiment and simulation. Gray represents the error against the training data while red represents the error against the validation data. For the final reweighting the value of theta which represents the minimum of $\mathcal{X}^2$ against the validation set is recommended if the ensemble preservation (blue) is sufficient.

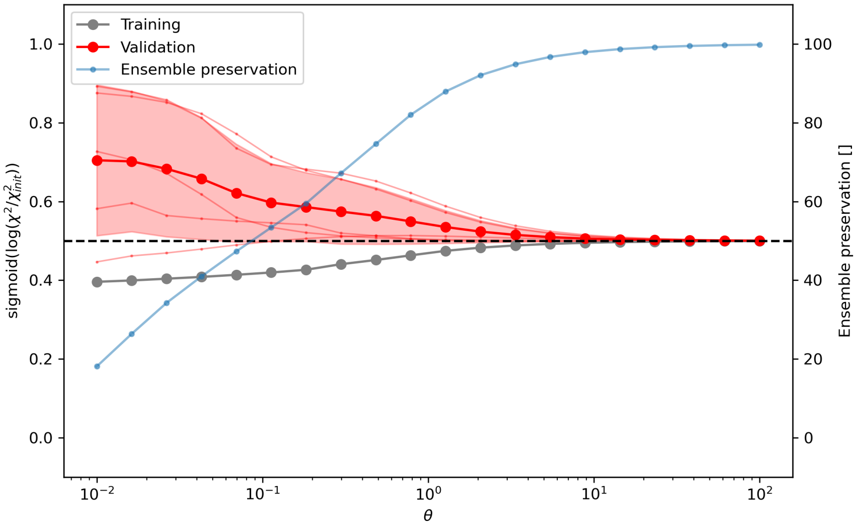

Figure S5: Cross validation of the Zwitterion reweighting using prior weights from calculations with Amber ff14SB. The x-axis shows the chosen value of theta while the y-axis shows the error between experiment and simulation. Gray represents the error against the training data while red represents the error against the validation data. For the final reweighting the value of theta which represents the minimum of $\mathcal{X}^2$ against the validation set is recommended if the ensemble preservation (blue) is sufficient.

In this specific case, with prior weights obtained from the Amber ff14SB force field, a validation-score curve with no minimum except on the boundary points was found. Therefore, we interpret this result as if no suggestion for theta was found. The ensemble preservation seems well-behaving with a preservation score of around 50 at a theta of 0.1. Therefore we find it to be acceptable to use a theta of 0.2 to allow a comparison with the results obtained using the initial weights of the other systems.

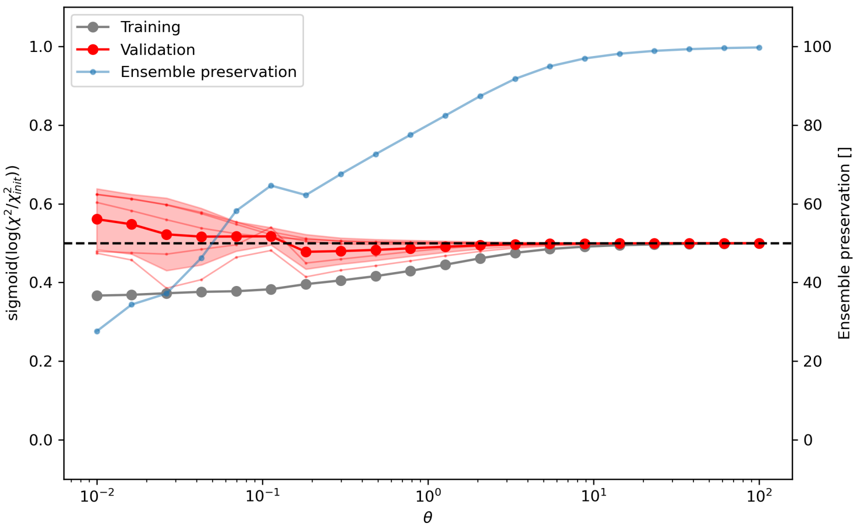

Figure S6: Cross validation of the Zwitterion reweighting using prior weights from calculations with MOPAC PM7. The x-axis shows the chosen value of theta while the y-axis shows the error between experiment and simulation. Gray represents the error against the training data while red represents the error against the validation data. For the final reweighting the value of theta which represents the minimum of $\mathcal{X}^2$ against the validation set is recommended if the ensemble preservation (blue) is sufficient.

4 Chignolin

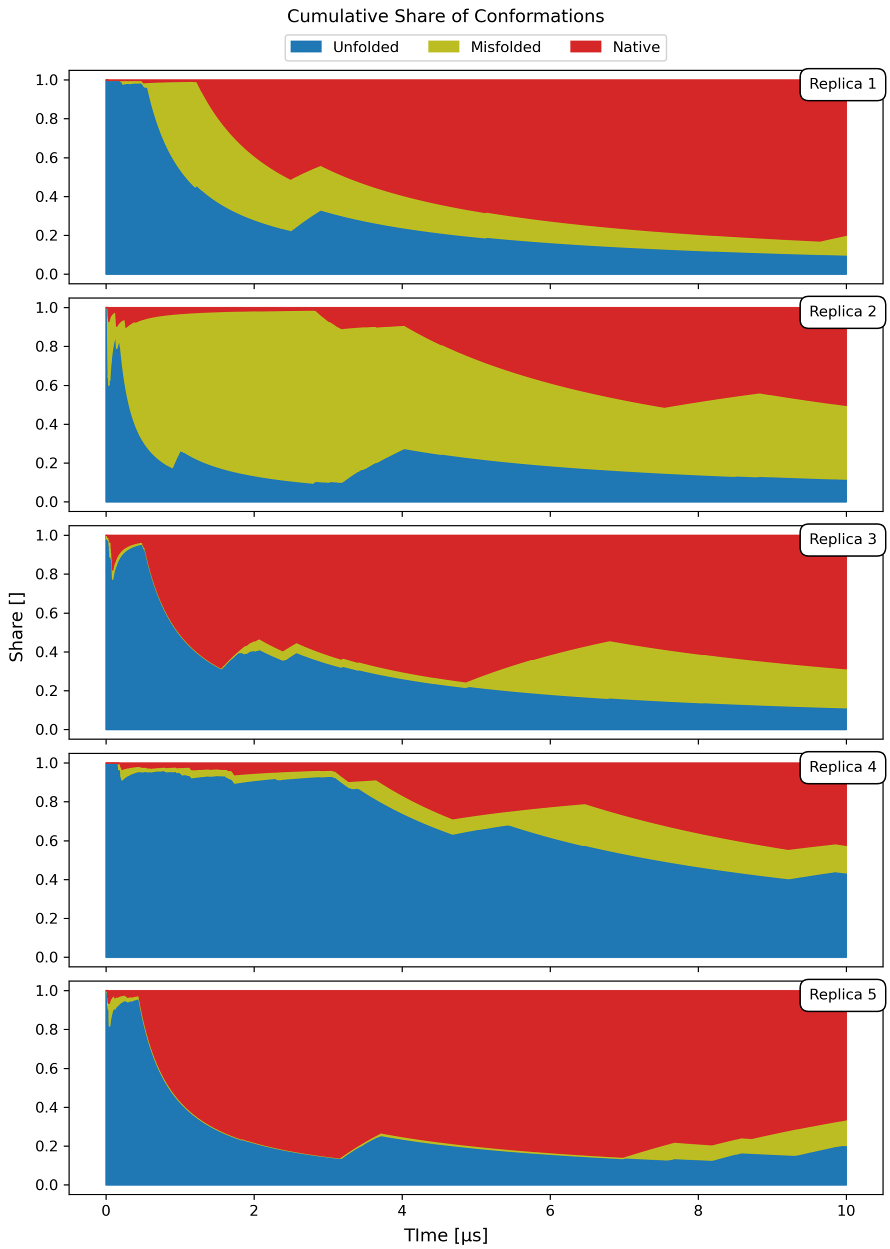

Figure S7: The cumulative share of the three main conformations during the simulated period. The simulation of all five replica started from an unfolded starting structure. Therefore, in the beginning the unfolded conformation is dominating. Fast folding can be observed within the first nanosecond in all replicas except replica 4.

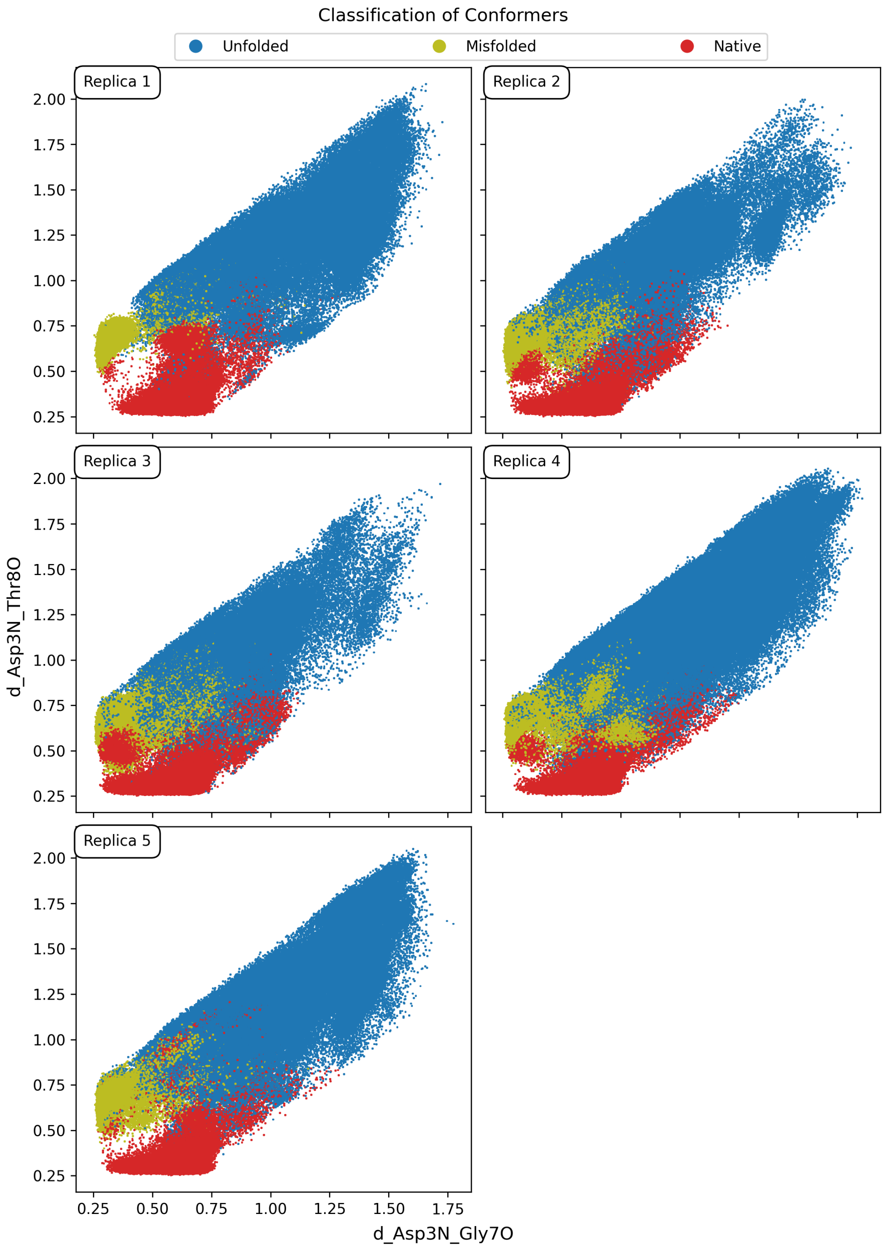

Figure S8: Each frame of the sparse trajectory has been classified to show either a native, misfolded or unfolded conformation. The x-axis shows the distance between atom N of residue 3 (ASP) and atom O of residue 7 (GLY) of each frame. The y-axis shows the distance between atom N of residue 3 (ASP) and atom O of residue 8 (THR).

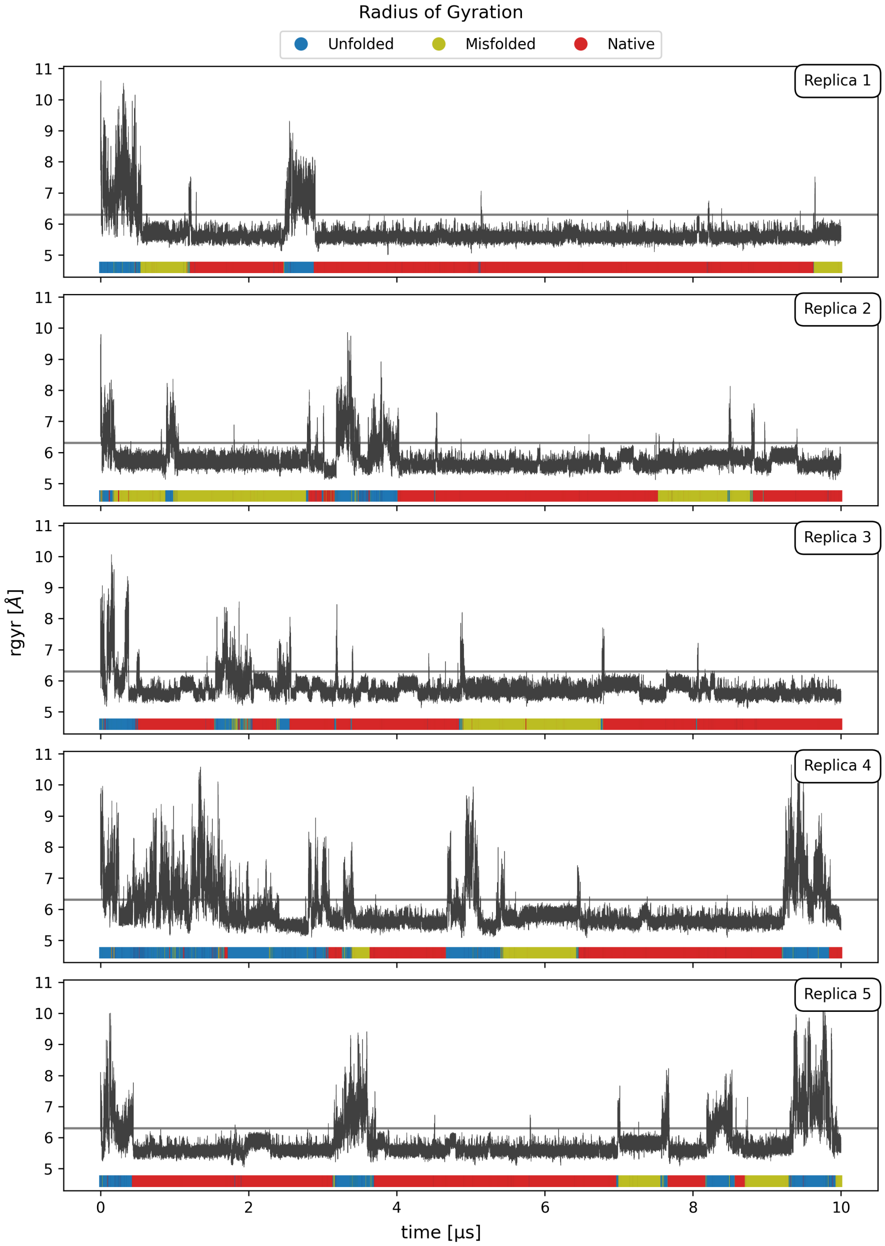

Figure S9: The radius of gyration (rgyr) is used as metric wether or not the protein is considered to be folded. The cutoff is set to 6.3 Å.

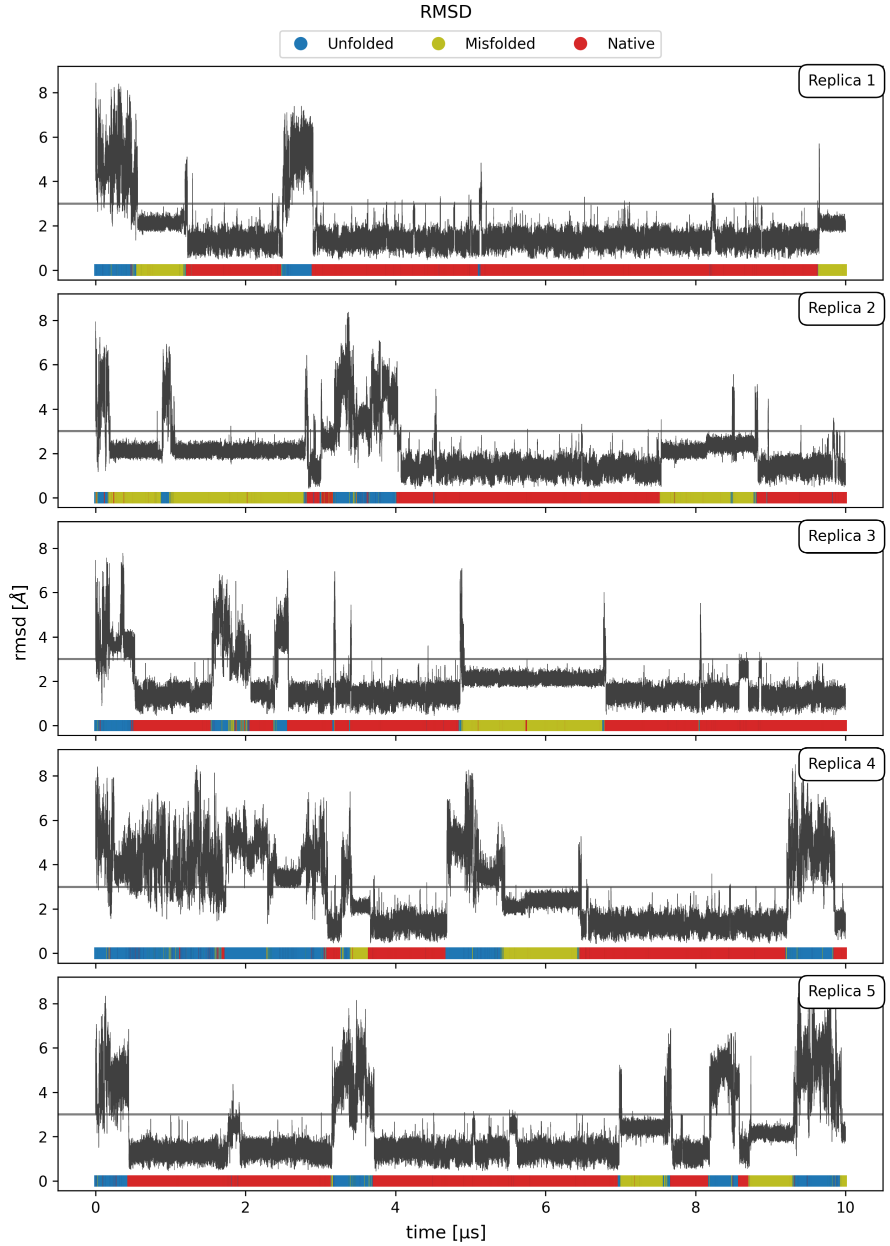

Figure S10: The RMSD is used as metric wether or not the protein is considered to be folded. The cutoff is set to 3.0 Å.

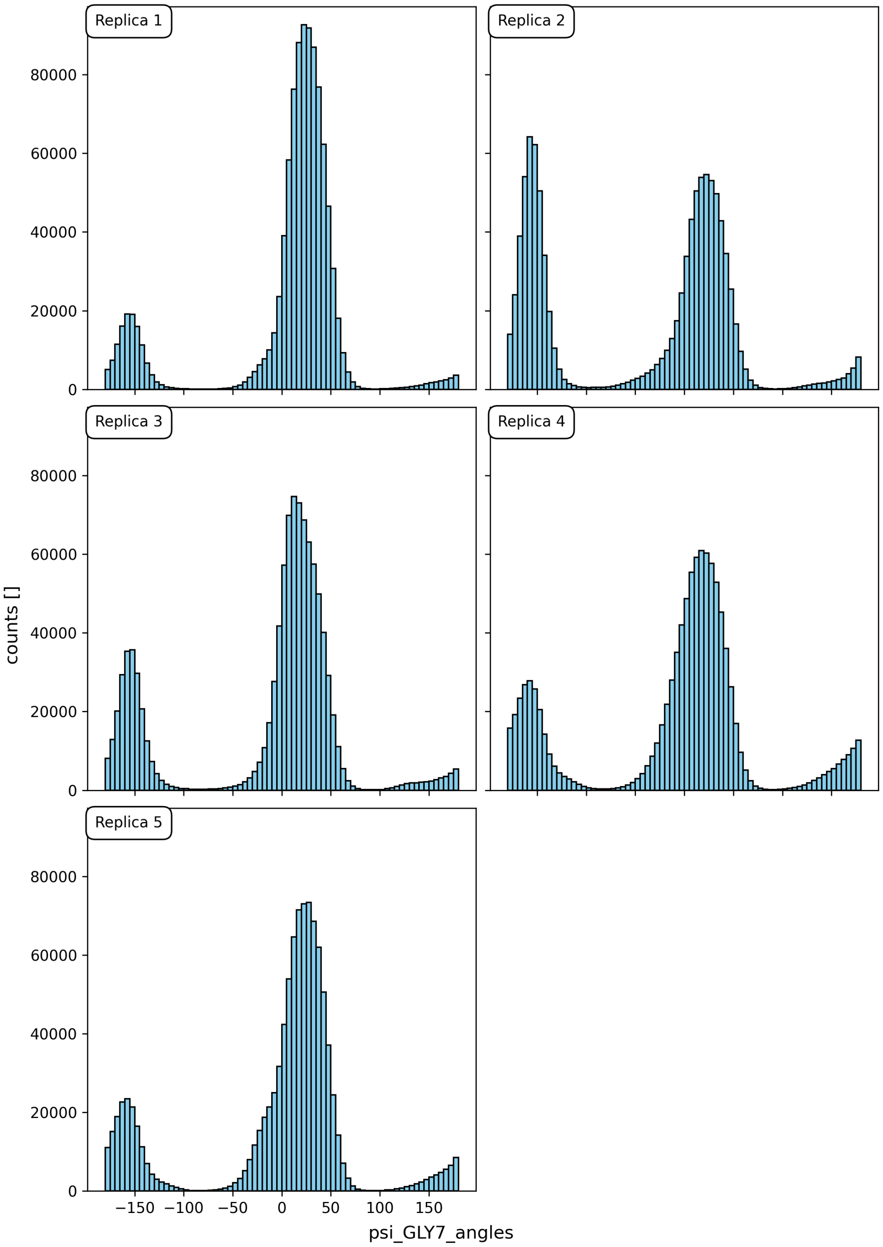

Figure S11: The classification whether the protein is natively folded or misfolded is done using the psi angle of residue 7 (GLY). The protein is assumed to be natively folded, when this dihedral is between -75 and 95 degree.

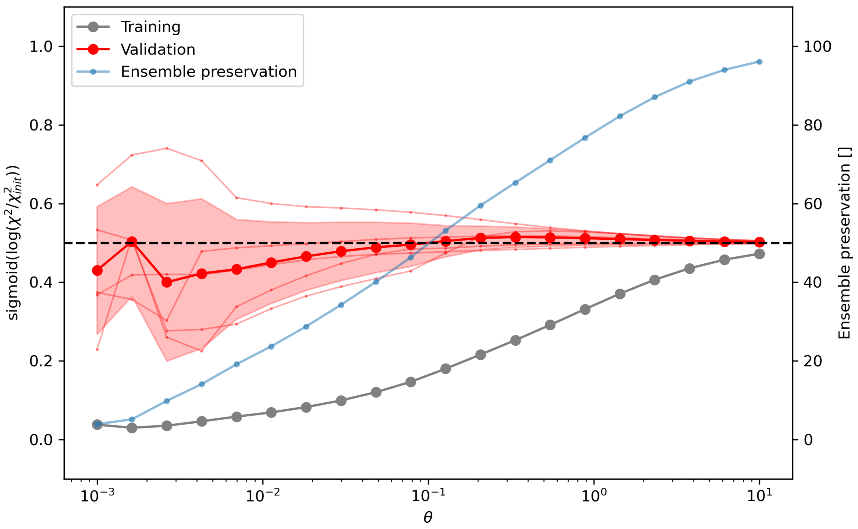

Figure S12: Replica 1 of the five-fold cross validation of the Chignolin reweighting. The x-axis shows the chosen value of theta while the y-axis shows the error between experiment and simulation. Gray represents the error against the training data while red represents the error against the validation data. For the final reweighting the value of theta which represents the minimum of $\mathcal{X}^2$ against the validation set is recommended if the ensemble preservation (blue) is sufficient.

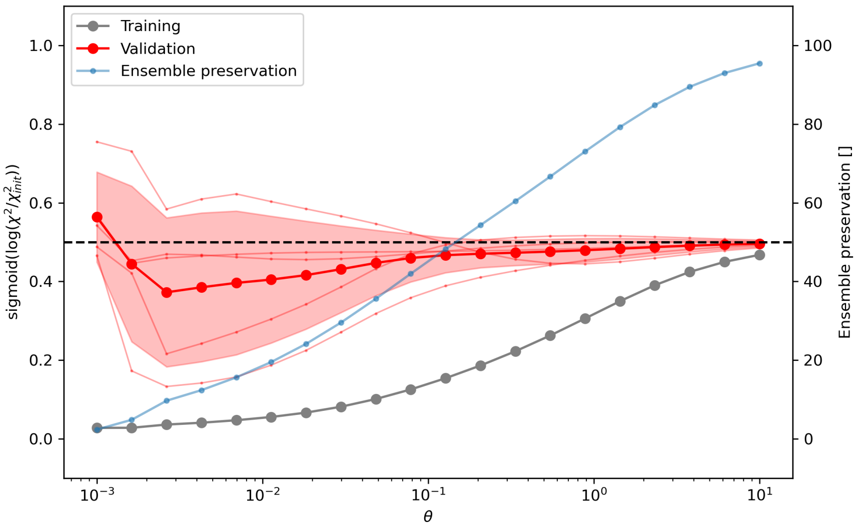

Figure S13: Replica 2 of the five-fold cross validation of the Chignolin reweighting. The x-axis shows the chosen value of theta while the y-axis shows the error between experiment and simulation. Gray represents the error against the training data while red represents the error against the validation data. For the final reweighting the value of theta which represents the minimum of $\mathcal{X}^2$ against the validation set is recommended if the ensemble preservation (blue) is sufficient.

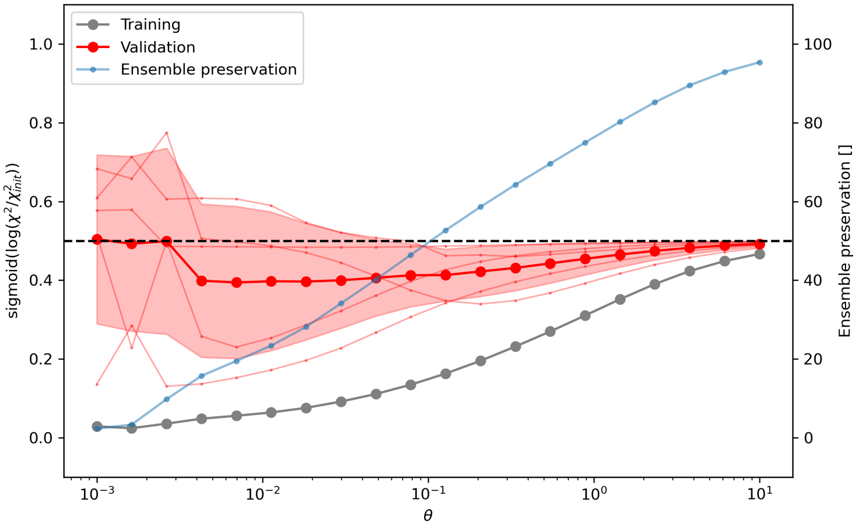

Figure S14: Replica 3 of the five-fold cross validation of the Chignolin reweighting. The x-axis shows the chosen value of theta while the y-axis shows the error between experiment and simulation. Gray represents the error against the training data while red represents the error against the validation data. For the final reweighting the value of theta which represents the minimum of $\mathcal{X}^2$ against the validation set is recommended if the ensemble preservation (blue) is sufficient.

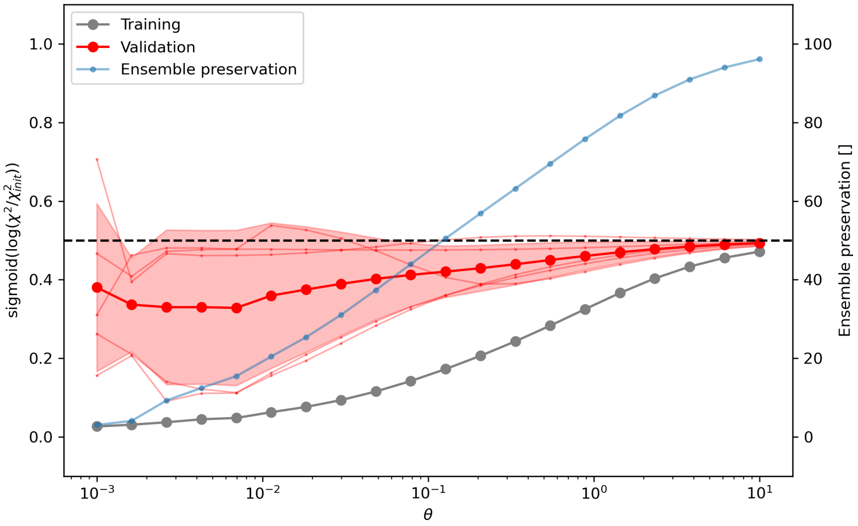

Figure S15: Replica 4 of the five-fold cross validation of the Chignolin reweighting. The x-axis shows the chosen value of theta while the y-axis shows the error between experiment and simulation. Gray represents the error against the training data while red represents the error against the validation data. For the final reweighting the value of theta which represents the minimum of $\mathcal{X}^2$ against the validation set is recommended if the ensemble preservation (blue) is sufficient.

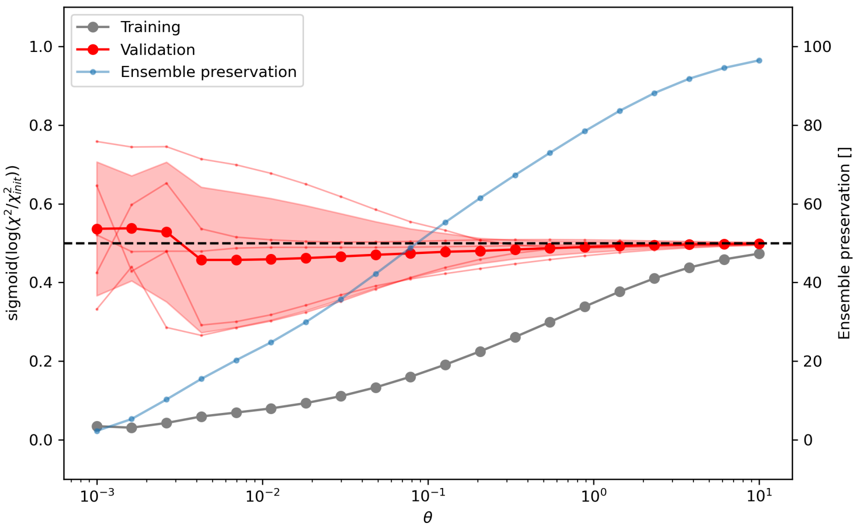

Figure S16: Replica 5 of the five-fold cross validation of the Chignolin reweighting. The x-axis shows the chosen value of theta while the y-axis shows the error between experiment and simulation. Gray represents the error against the training data while red represents the error against the validation data. For the final reweighting the value of theta which represents the minimum of $\mathcal{X}^2$ against the validation set is recommended if the ensemble preservation (blue) is sufficient.

Annotation: In the raw data, the five replica were counted starting from 6 up to 10. In the manuscript, the five replica were chronologically renamed to start from 1 and end with 5.

5 References

[1] A. Chan, H. Silva, S. Lim, T. Kozuno, A. R. Mahmood, M. White, "Greedification operators for policy optimization: investigating forward and reverse KL divergences," *J. Mach. Learn. Res.* **2022**, *23*, 1–79.

[2] L. Vaitl, K. A. Nicoli, S. Nakajima, P. Kessel, "Gradients should stay on path: better estimators of the reverse- and forward KL divergence for normalizing flows," *Machine Learning: Science and Technology* **2022**, *3*, 045006.

[3] M. Shen, N. Diamant, On KL Divergence in Discrete Spaces, Accessed on 2024-08-14, **2022**, `https://argmax.blog/posts/kl-discrete/`.
